# Structure-Aware Progressive Multi-Modal Fusion Network for RGB-T Crack Segmentation

**DOI:** 10.3390/jimaging11110384

**Published:** 2025-11-01

**Authors:** Zhengrong Yuan, Xin Ding, Xinhong Xia, Yibin He, Hui Fang, Bo Yang, Wei Fu

**Affiliations:** 1 Hunan Architectural Design Institute Group Co., Ltd., Changsha 410208, China; yuanzr@hnadi.com.cn (Z.Y.); xiaxh@hnadi.com.cn (X.X.); fangh@hnadi.com.cn (H.F.); yangbo@hnadi.com.cn (B.Y.); 2 School of Artificial Intelligence and Robotics, Hunan University, Changsha 410012, China; dingxin_2002@163.com; 3 College of Computer Science and Electronic Engineering, Hunan University, Changsha 410082, China; fuwei@hnu.edu.cn

**Keywords:** crack segmentation, deep learning, gate control attention, feature fusion

## Abstract

Crack segmentation in images plays a pivotal role in the monitoring of structural surfaces, serving as a fundamental technique for assessing structural integrity. However, existing methods that rely solely on RGB images exhibit high sensitivity to light conditions, which significantly restricts their adaptability in complex environmental scenarios. To address this, we propose a structure-aware progressive multi-modal fusion network (SPMFNet) for RGB-thermal (RGB-T) crack segmentation. The main idea is to integrate complementary information from RGB and thermal images and incorporate structural priors (edge information) to achieve accurate segmentation. Here, to better fuse multi-layer features from different modalities, a progressive multi-modal fusion strategy is designed. In the shallow encoder layers, two gate control attention (GCA) modules are introduced to dynamically regulate the fusion process through a gating mechanism, allowing the network to adaptively integrate modality-specific structural details based on the input. In the deeper layers, two attention feature fusion (AFF) modules are employed to enhance semantic consistency by leveraging both local and global attention, thereby facilitating the effective interaction and complementarity of high-level multi-modal features. In addition, edge prior information is introduced to encourage the predicted crack regions to preserve structural integrity, which is constrained by a joint loss of edge-guided loss, multi-scale focal loss, and adaptive fusion loss. Experimental results on publicly available RGB-T crack detection datasets demonstrate that the proposed method outperforms both classical and advanced approaches, verifying the effectiveness of the progressive fusion strategy and the utilization of the structural prior.

## 1. Introduction

Human transportation, habitation, and production are inseparable from a large number of man-made structures such as buildings, roads, and bridges. As time passes, these structures suffer from structural damages, which are usually indicated by surface cracks. Therefore, to ensure the safety of people’s lives and property, it is necessary to regularly monitor the surface cracks of man-made structures. Compared with on-site manual inspection, automatic crack segmentation methods based on UAV images are low cost, low risk, and can monitor inaccessible areas.

Semantic segmentation is the critical technique for crack segmentation. It is a task of dense pixel-wise classification, where each pixel in an image is assigned a corresponding semantic category label [1]. In the field of infrastructure maintenance, semantic segmentation-based crack extraction draws increasing attention in various civil engineering scenarios such as building inspection [2], bridge monitoring [3], tunnel safety evaluation [4], and road surface analysis [5]. In recent years, deep learning technologies, especially convolutional neural networks (CNNs), have brought revolutionary advancements to the field of semantic segmentation. A series of milestone architectures have emerged, including Fully Convolutional Networks (FCNs) [6], U-Net [7], and DeepLab V3+ [8], all of which have achieved remarkable results in large-scale RGB image semantic segmentation tasks. Inspired by these successes, researchers gradually introduce these advanced techniques in the field of crack segmentation. Most methods tend to adopt convolutional architectures. For example, the DeepCrack [9] utilizes hierarchical multi-scale feature extraction methods to capture fine structural crack details. APF-Net [10] integrates a progressive fusion (PF) module and a hybrid multiple attention (HMA) mechanism, and this technique has been proposed for pavement crack segmentation.

Transformer-based approaches have also demonstrated strong performance. CrackFormer-II [11] employs local self-attention and attention-guided skip connections to improve both global context modeling and fine detail segmentation. CrackSegNet [12] adopts dilated convolutions and multi-scale pooling within a modular architecture, addressing class imbalance with an optimized loss function. A two-stage method proposed by Liu combines Mask R-CNN for detection and DeepLabV3+ for fine segmentation in bridge crack images [13].

To segment thin and low-contrast cracks more accurately, StripCuts [14] formulates segmentation as an optimization problem, leveraging volumetric dynamic programming and crack linearization. Li et al. [15] introduced a physically informed method that incorporates dynamic snake convolution and cross-correlation constraints to enhance continuity in road crack segmentation. Furthermore, Yoon et al. [16] developed a comprehensive polygon-annotated crack dataset with diverse crack types, serving as a robust benchmark for semantic and instance segmentation.

In addition, several methods are developed for specific environments. For example, Sun et al. proposed a hybrid model combining an EfficientNet-enhanced YOLOX for crack identification and an UNETR++ for fine segmentation [17] for tunnel environments. Similarly, ISTD-CrackNet [18] uses a hierarchical Transformer with multi-angle strip convolution and dynamic upsampling to maintain crack continuity and sharp edges. In complex mining environments, Wang et al. [19] employed mid-level semantic features and multi-scale attention fusion within a DeepLabV3+-based network to improve segmentation under noisy backgrounds. To address the challenge of detecting fine linear fatigue cracks in metallic aero-engine components, Si et al. [20] developed a VGG-16-based U-shaped FCN model integrating CBAM and advanced loss functions, achieving significant improvements in segmentation performance. Several other end-to-end architectures have also been proposed. Wang et al. [21] introduced a U-Net variant incorporating multi-layer feature fusion, residual blocks with pointwise convolution, and maximum unpooling to enhance thin-crack edge detail. DCNCrack [22] integrates deformable convolution into CNNs to improve adaptive spatial feature aggregation and long-range dependence. MorFormer [23] combines background morphology learning and morphology-aware attention to suppress noise and capture crack topology. Semi-CSN [24] adopts a semi-supervised training strategy with multi-scale attention fusion and dynamic pseudo-labeling to leverage unlabeled data effectively. In the domain of weakly supervised learning, CrackCLIP [25] utilizes vision-language pre-training (CLIP) and language prompts to guide semantic understanding and pseudo-label generation.

However, under challenging conditions such as rainy weather, haze, or low-light environments, crack detection and segmentation methods that rely solely on RGB images exhibit limited performance. Moreover, approaches that depend exclusively on infrared images may hinder the model’s ability to distinguish cracks from other surface defects (e.g., stains, dust, or corrosion), thereby leading to false positives or missed detections. To address these issues, RGB-T crack segmentation techniques [26] have been proposed in recent years because thermal images do not depend on light conditions and are complementary to RGB images [27]. Some RGB-T semantic segmentation networks have been developed to achieve good results in other fields. It means that multi-modal information can promote the stability and accuracy of semantic segmentation. For instance, Ha et al. [28] pioneered this approach with the introduction of MFNet, which utilizes two independent but structurally identical encoders to extract modality-specific features, followed by a unified fusion strategy in the decoder for cross-modal integration. Since the introduction of MFNet, numerous models have been developed under the symmetric framework. Zhang et al. [29] proposed ABMDRNet, which incorporates a dedicated sub-network to reduce the domain gap between RGB and thermal features during the fusion process, thereby enabling more effective joint representation learning using shared operations. Similarly, Zhou et al. [30] introduced GMNet, which decomposes features into two semantic levels and applies distinct fusion modules accordingly while employing multi-label supervision to guide training. Zhou et al. [31] decoupled the feature fusion process from the encoder and decoder stages, extracting global contextual information from high-level fused features via a parallel convolutional structure to enhance decoding performance. More recently, to further explore cross-modal complementarities and morphological diversity, MMSMCNet [32] was proposed. It integrates modal memory sharing with multi-scale morphological guidance, introducing a novel decoder composed of contour, skeleton, and morphological complementary modules, and achieves superior performance through a multi-unit-based complementary supervision strategy. Beyond fusion strategy innovations, some models have begun focusing on enhancing the decoding process itself. For example, the Shape and Semantic Enhancements Module (SASEM) [33] proposes a dual-branch decoder structure that separately reinforces shape and semantic representations using signed distance maps and channel-level enhancement, which effectively improves feature recovery and object boundary integrity. Furthermore, UTFNet [34] introduces an uncertainty-guided RGB-T fusion strategy by quantifying the modality confidence of each input through evidential theory. It dynamically adjusts the fusion based on illumination evidence and multi-scale reliability, enabling more trustworthy cross-modal integration under varied real-world conditions. In addition, DHFNet [35] proposes a hierarchical fusion approach that decouples global and local features from each modality. Through modules such as lightweight global self-attention (LGSA), cross-modal deformable convolution (CMDC), and long-distance feature fusion (CMLFF), it effectively addresses feature misalignment and redundancy, achieving fine-grained fusion across different levels. Zhao et al. [36] propose OpenRSS, an open-vocabulary RGB-T semantic segmentation method that efficiently fuses RGB and thermal information, achieving significant improvements in multiple benchmarks. Liu et al. [37] introduce IQSeg, which enhances RGB-T segmentation accuracy by addressing implicit alignment and refining query-based predictions. Liu et al. [38] present MiLNet, which is a module-free network that integrates multiplex feature interactions to improve RGB-T semantic segmentation performance.

These indicate that the fusion of multi-modal information can promote the performance of semantic segmentation, especially in terms of the stability and accuracy. To better leverage the complementary information from both modalities, an effective multi-modal feature fusion mechanisms [39] should be carefully designed. Moreover, we note that cracks often exhibit tiny, intermittent, and irregular edges, making it difficult to accurately segment cracks without effective edge features. To this end, we propose a crack detection method based on the structure-adaptive fusion of RGB and thermal images. The main contributions of this work are as follows:

- An end-to-end edge-guided progressive multi-modal segmentation network is proposed in this work. The developed framework is designed to jointly leverage both RGB and thermal infrared information while incorporating structural priors. By embedding edge-aware supervision throughout the decoding process, the overall accuracy and robustness of crack segmentation are significantly enhanced, effectively addressing the boundary blurring issue commonly encountered in conventional approaches.
- A progressive multi-modal feature fusion strategy is designed to hierarchically integrate cross-modal features. For shallow-level features characterized by rich spatial details, a Gated Control Attention (GCA) module is introduced to dynamically recalibrate modal contributions through a gating mechanism, thereby enhancing texture perception in local crack regions. For deep-level features carrying high-level semantics, an Attention Feature Fusion (AFF) module is employed to align and complement cross-modal representations via joint local–global attention, effectively strengthening semantic consistency in structural prediction.
- A structure-prior-guided segmentation prediction strategy is proposed, which utilizes edge prediction consistency constraints to preserve the original structural characteristics in segmentation results. By formulating a joint optimization objective comprising edge-guided loss, multi-scale focal loss, and adaptive fusion loss, the structural integrity and boundary accuracy of the final predictions are significantly improved.

In Section 2, we comprehensively describe the proposed approach, while Section 3 presents empirical evaluations and Section 4 concludes this paper.

## 2. Proposed Method

In this section, we first introduce the framework of the proposed method. Then, detailed descriptions about the progressive multi-modal feature fusion stage and the structure-guided crack segmentation stage, as well as developed modules, are demonstrated. Finally, the edge-guided loss function is coupled with a classic loss function to employ the bias of crack structures.

**Figure 1:**
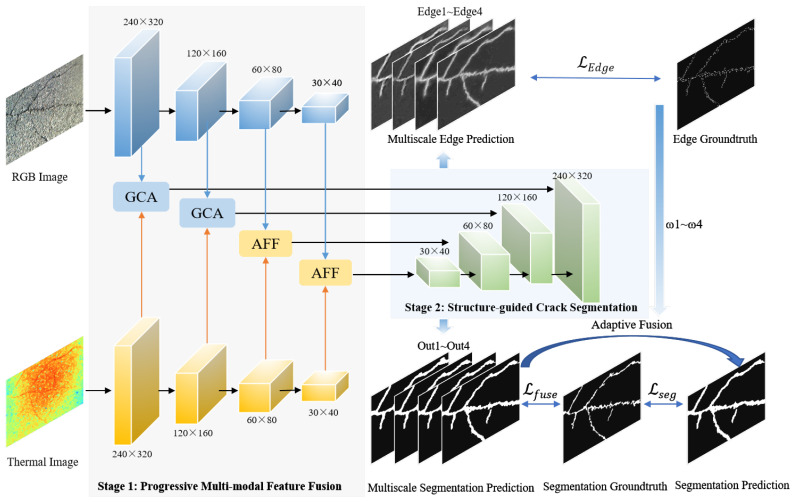
The structure of the proposed SPMFNet.

The proposed SPMFNet (Structure-aware Progressive Multi-modal Fusion Network for RGB-T Crack Segmentation) method mainly consists of two stages, as shown in Figure 1. When RGB and thermal images are input, the dual-branch encoder extracts multi-level semantic features from both modalities using a four-stage hierarchy. To enable stage-specific fusion, the encoder employs a Gated Control Attention (GCA) module in the first two layers for fine-grained cross-modal interaction. Furthermore, an Attentional Feature Fusion (AFF) module in the last two layers is used to enhance semantic alignment at deeper levels. These adaptively fused features are subsequently passed to the decoder, which performs progressive upsampling with skip connections to recover spatial resolution. The decoder further integrates edge-aware information and outputs pixel-level segmentation maps refined by an edge-guided supervision strategy, thereby enabling accurate and robust crack detection under complex environmental conditions.

### 2.1. Progressive Multi-Modal Feature Fusion

The proposed multi-modal crack detection network employs a dual-branch symmetric encoder to independently extract features from RGB and thermal (T) images through parallel paths of four hierarchical convolutional layers. To fully exploit the complementary characteristics of these modalities—namely the structural richness of RGB and the thermal sensitivity of T—the encoder integrates distinct fusion strategies adapted to semantic depth. In the first two shallow encoding stages, a Gated Control Attention (GCA) module dynamically performs spatially adaptive cross-modal fusion via hierarchical gated attention, which selectively modulates modality contributions based on the spatial scene context. This mechanism enhances local crack perception by suppressing redundancy and emphasizing modality-specific structural details. In the deeper third and fourth stages, an Attentional Feature Fusion (AFF) module is employed, combining both channel-wise and spatial attention branches to selectively aggregate high-level semantic features and improve semantic alignment across modalities. This depth-aware, staged fusion strategy enables the effective preservation of fine spatial details in shallow layers while ensuring semantic consistency at deeper layers, thereby enhancing the robustness and accuracy of crack representation under complex imaging conditions. In the progressive multi-modal feature fusion stage, a novel combination of techniques was employed to achieve a high-quality fusion of RGB and thermal modal features.

Overall, the encoder architecture leverages a progressive fusion strategy that aligns well with the hierarchical nature of feature extraction. By combining modality-specific low-level cues with jointly refined high-level semantics, the encoder ensures the preservation of detailed spatial information while enhancing the representation of complex crack structures. By employing GCA in early encoding layers and AFF in deeper stages, the network benefits from both fine-grained spatial adaptivity and high-level semantic selectivity. This hierarchical fusion scheme ensures that the encoder captures crack-related cues with varying spatial and contextual granularity, thereby laying a solid foundation for the subsequent edge-guided decoding and final segmentation prediction.

#### 2.1.1. Gate Control Attention Module

To enhance the interaction and integration of modality-specific features in shallow layers, a Gate Control Attention (GCA) module is designed and deployed in the encoder. GCA is specifically constructed to perform cross-modal interaction and dynamic feature fusion between RGB and thermal image representations.

**Figure 2:**
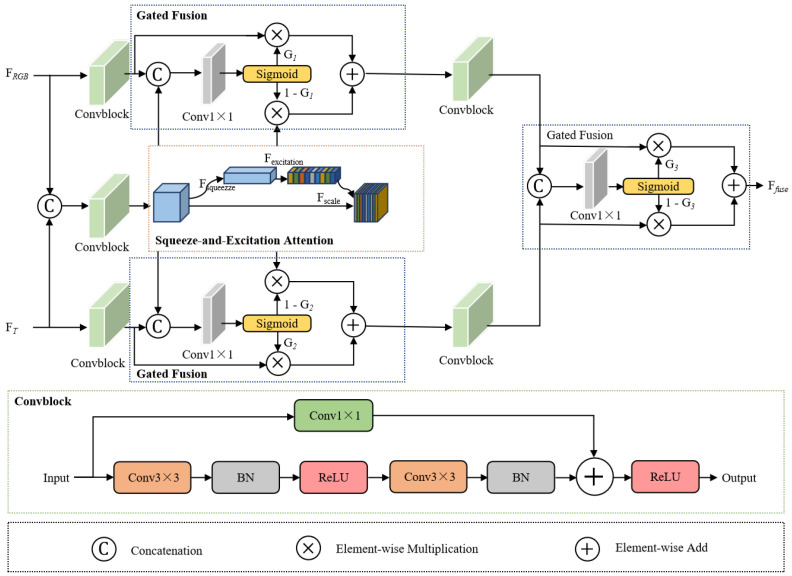
The structure of the proposed GCA.

The GCA is designed to perform cross-modal interaction and dynamic feature fusion. For each layer, the features extracted from the RGB and thermal branches are concatenated along the channel dimension to construct the input for fusion. The structure of the GCA is illustrated in Figure 2. Let $F_{\mathrm{RGB}}^{i}$ and $F_{T}^{i}$ denote the input features of the RGB and thermal branches at the *i*-th (i∈{1,2}) layer, respectively. The inputs $F_{\mathrm{RGB}}^{i}$ and $F_{T}^{i}$ are respectively processed by a Convblock module to generate enhanced features $F_{\mathrm{RGBC}}^{i}$ and $F_{\mathrm{TC}}^{i}$, which is formulated as follows:

(1) $$F_{\mathrm{RGBC}}^{i}=\mathrm{ReLU}\left({\mathrm{Conv}}_{1\times 1}\left(F_{\mathrm{RGB}}^{i}\right)+\mathrm{BasicConv}\left(F_{\mathrm{RGB}}^{i}\right)\right),\;i=1,2.$$

(2) $$\mathrm{BasicConv}\left(F_{\mathrm{RGB}}^{i}\right)=\mathrm{BN}\left({\mathrm{Conv}}_{3\times 3}\left(\mathrm{ReLU}\left(\mathrm{BN}\left({\mathrm{Conv}}_{3\times 3}\left(F_{\mathrm{RGB}}^{i}\right)\right)\right)\right)\right),\;i=1,2.$$

where BN is batch normalization, ${\mathrm{Conv}}_{3\times 3}$ denotes a learnable 3×3 convolution operation, and ReLU is the rectified linear unit activation function.

(3) $$F_{\mathrm{TC}}^{i}=\mathrm{ReLU}\left({\mathrm{Conv}}_{1\times 1}\left(F_{T}^{i}\right)+\mathrm{BasicConv}\left(F_{T}^{i}\right)\right),\;i=1,2.$$

(4) $$\mathrm{BasicConv}\left(F_{T}^{i}\right)=\mathrm{BN}\left({\mathrm{Conv}}_{3\times 3}\left(\mathrm{ReLU}\left(\mathrm{BN}\left({\mathrm{Conv}}_{3\times 3}\left(F_{T}^{i}\right)\right)\right)\right)\right),\;i=1,2.$$

By sequentially applying feature concatenation, Convblock, and the squeeze-and-excitation operation proposed by Hu et al. [40], the feature representation $F_{\mathrm{SE}}^{i}$ is obtained. The computation is formulated as shown below:

(5) $$F_{\mathrm{SE}}^{i}=\mathrm{SE}\left(\mathrm{ReLU}\left({\mathrm{Conv}}_{1\times 1}\left(F_{\mathrm{RGB}}^{i}\right)+\mathrm{BasicConv}\left(\mathrm{Concat}\left(F_{\mathrm{RGB}}^{i},F_{T}^{i}\right)\right)\right)\right)$$

where SE is the squeeze-and-excitation operation.

The gated fusion between $F_{\mathrm{RGBC}}^{i}$ and $F_{\mathrm{SE}}^{i}$ is formulated as follows:

(6) $$G_{1}^{i}=\sigma \left({\mathrm{Conv}}_{1\times 1}\left(\mathrm{Concat}\left(F_{\mathrm{RGBC}}^{i},F_{\mathrm{SE}}^{i}\right)\right)\right),\;i=1,2.$$

where ${\mathrm{Conv}}_{1\times 1}$ denotes the learnable convolution operation with kernel size 1×1, σ(·) is the sigmoid activation function, and Concat(·,·) represents the concatenation operation along the channel dimension. The output $G_{1}^{i}$ denotes the spatial attention weights.

The first fused feature output $F_{\mathrm{fuse}1}^{i}$ is defined as shown below:

(7) $$F_{\mathrm{fuse}1}^{i}=G_{1}^{i}\cdot F_{\mathrm{RGBC}}^{i}+\left(1-G_{1}^{i}\right)\cdot F_{\mathrm{SE}}^{i},\;i=1,2.$$

Then, the gated fusion between $F_{\mathrm{TC}}^{i}$ and $F_{\mathrm{SE}}^{i}$ is formulated as follows:

(8) $$G_{2}^{i}=\sigma \left({\mathrm{Conv}}_{1\times 1}\left(\mathrm{Concat}\left(F_{\mathrm{TC}}^{i},F_{\mathrm{SE}}^{i}\right)\right)\right),\;i=1,2.$$

The second fused feature output $F_{\mathrm{fuse}2}^{i}$ is defined as shown below:

(9) $$F_{\mathrm{fuse}2}^{i}=G_{2}^{i}\cdot F_{\mathrm{TC}}^{i}+\left(1-G_{2}^{i}\right)\cdot F_{\mathrm{SE}}^{i},\;i=1,2.$$

After that, $F_{\mathrm{fuse}1}^{i}$ and $F_{\mathrm{fuse}2}^{i}$ are, respectively, passed through a Convblock module to generate the refined features $F_{\mathrm{fuseC}1}^{i}$ and $F_{\mathrm{fuseC}2}^{i}$. The calculation formulas are as follows:

(10) $$F_{\mathrm{fuseC}1}^{i}=\mathrm{ReLU}\left({\mathrm{Conv}}_{1\times 1}\left(F_{\mathrm{fuse}1}^{i}\right)+\mathrm{BasicConv}\left(F_{\mathrm{fuse}1}^{i}\right)\right),\;i=1,2.$$

(11) $$\mathrm{BasicConv}\left(F_{\mathrm{fuse}1}^{i}\right)=\mathrm{BN}\left({\mathrm{Conv}}_{3\times 3}\left(\mathrm{ReLU}\left(\mathrm{BN}\left({\mathrm{Conv}}_{3\times 3}\left(F_{\mathrm{fuse}1}^{i}\right)\right)\right)\right)\right),\;i=1,2.$$

(12) $$F_{\mathrm{fuseC}2}^{i}=\mathrm{ReLU}\left({\mathrm{Conv}}_{1\times 1}\left(F_{\mathrm{fuse}2}^{i}\right)+\mathrm{BasicConv}\left(F_{\mathrm{fuse}2}^{i}\right)\right),\;i=1,2.$$

(13) $$\mathrm{BasicConv}\left(F_{\mathrm{fuse}2}^{i}\right)=\mathrm{BN}\left({\mathrm{Conv}}_{3\times 3}\left(\mathrm{ReLU}\left(\mathrm{BN}\left({\mathrm{Conv}}_{3\times 3}\left(F_{\mathrm{fuse}2}^{i}\right)\right)\right)\right)\right),\;i=1,2.$$

The gated fusion between $F_{\mathrm{fuseC}1}^{i}$ and $F_{\mathrm{fuseC}2}^{i}$ is formulated as follows:

(14) $$G_{3}^{i}=\sigma \left({\mathrm{Conv}}_{1\times 1}\left(\mathrm{Concat}\left(F_{\mathrm{fuseC}1}^{i},F_{\mathrm{fuseC}2}^{i}\right)\right)\right),\;i=1,2.$$

The final output of the fused feature $F_{\mathrm{fuse}}^{i}$ (i∈{1,2}) is computed using a weighted combination of elements:

(15) $$F_{\mathrm{fuse}}^{i}=G_{3}^{i}\cdot F_{\mathrm{fuseC}1}^{i}+\left(1-G_{3}^{i}\right)\cdot F_{\mathrm{fuseC}2}^{i},\;i=1,2.$$

The key innovation of GCA lies in its gating mechanism, which allows the network to dynamically adjust the fusion ratio of the two modalities based on spatial and semantic contexts. By learning position-specific gating weights, the module can adaptively balance modality contributions, enabling the network to fully exploit complementary information from both RGB and thermal sources. This result in a more expressive and context-aware multi-modal feature representation, which is critical for precise crack segmentation under diverse environmental conditions.

In addition to this core gating mechanism, the GCA module also integrates Squeeze-and-Excitation Attention to refine the coarse fused features. It implements a Squeeze-and-Excitation mechanism that extracts global channel statistics through average pooling and applies adaptive channel weighting via a two-layer bottleneck structure. This allows the model to enhance informative channels and suppress irrelevant ones, improving the quality of the joint representation.

**Figure 3:**
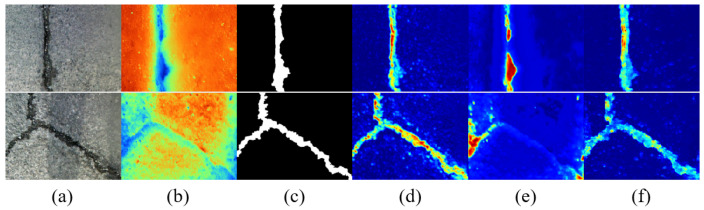
Comparison features of the GCA module before and after fusion: (**a**) original RGB images, (**b**) original thermal images, (**c**) label images, (**d**) RGB features before fusion, (**e**) thermal features before fusion, and (**f**) fused features.

To enable fine-grained interactions, the GCA module integrates a hierarchical gated fusion strategy. This strategy employs three independent GatedFusion blocks, each generating spatially adaptive gates via 1 × 1 convolutions followed by sigmoid activations. These gates modulate the contribution of each modality at the pixel level. The feature fusion process of the GCA module is conducted in three stages to progressively refine feature integration. The first two stages generate fusion maps between each modality and the interactive feature $F_{\mathrm{SE}}^{i}$, while the final stage aggregates the outputs of the preceding two stages to enhance the overall fusion quality. This multi-stage design facilitates precise feature alignment and fusion, allowing the model to capture subtle inter-modal dependencies. The final output is produced by aggregating the multiple gated results. Figure 3 presents the visualization of the GCA module before and after fusion on the asphalt pavement crack detection dataset.

Both SE-Block and CBAM are designed to enhance feature representation through attention mechanisms. SE-Block focuses on channel-wise recalibration via global pooling and channel weighting, while CBAM extends this idea by integrating spatial attention to simultaneously capture informative channels and spatial regions. GCA, on the other hand, not only incorporates the channel weighting concept of SE-Block but also introduces a gating mechanism and multi-feature fusion strategy, enabling dynamic cross-modal feature selection. Therefore, compared with SE-Block and CBAM, GCA shares the common goal of feature refinement but places greater emphasis on cross-modal fusion and adaptive feature integration.

#### 2.1.2. Attention Feature Fusion

**Figure 4:**
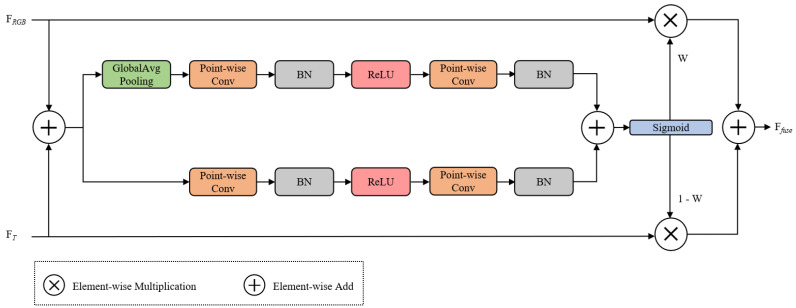
The structure of AFF.

In the last two stages of the encoder, we adopt the Attentional Feature Fusion (AFF) module proposed by Dai et al. [41], as illustrated in Figure 4. The module begins by performing an element-wise addition of RGB and thermal features to generate an initial fused representation. Then, AFF incorporates two parallel attention branches—local and global—to enhance the feature fusion process.

The local branch employs two successive 1 × 1 convolutions to capture nonlinear relationships between channels, while the global branch uses global average pooling to extract channel-wise contextual information, which is followed by a dimension restoration operation. The outputs from the two branches are summed and passed through a Sigmoid activation to form a fusion attention map, which dynamically evaluates the importance of each channel.

To adaptively fuse the input features from two sources, the AFF module computes a spatial attention weight $W^{i}$ based on both local and global contexts. Let $F_{\mathrm{RGB}}^{i}$ and $F_{T}^{i}$ denote the input features of the RGB and T branches at the *i*-th layer (i∈{3,4}), respectively. First, the input features $F_{\mathrm{RGB}}^{i}$ and $F_{T}^{i}$ are added element-wise to obtain $F_{add}^{i}$, which is computed as follows:

(16) $$F_{add}^{i}=F_{\mathrm{RGB}}^{i}+F_{T}^{i},\;i=3,4.$$

Then, $F_{add}^{i}$ passes through a local attention branch and a global attention branch. The local attention path applies two 1×1 convolutions and batch normalization with ReLU activation:

(17) $$F_{l}^{i}=\mathrm{BN}\left({\mathrm{Conv}}_{1\times 1}\left(\mathrm{ReLU}\left(\mathrm{BN}\left({\mathrm{Conv}}_{1\times 1}\left(F_{add}^{i}\right)\right)\right)\right)\right),\;i=3,4.$$

where BN is batch normalization, ${\mathrm{Conv}}_{1\times 1}$ denotes a learnable 1×1 convolution operation, and ReLU is the Rectified Linear Unit activation function.

While the global attention branch performs global average pooling followed by a similar two-layer bottleneck:

(18) $$F_{g}^{i}=\mathrm{BN}\left({\mathrm{Conv}}_{1\times 1}\left(\mathrm{ReLU}\left(\mathrm{BN}\left({\mathrm{Conv}}_{1\times 1}(\mathrm{GAP}\left(F_{add}^{i})\right)\right)\right)\right)\right),\;i=3,4.$$

where GAP(·) denotes global average pooling.

The outputs of both attention branches are aggregated to produce the fused attention descriptor $F_{lg}^{i}$:

(19) $$F_{lg}^{i}=F_{l}^{i}+F_{g}^{i},\;i=3,4.$$

Finally, a sigmoid activation is applied to obtain the attention weight $W^{i}$:

(20) $$W^{i}=\sigma \left(F_{lg}^{i}\right),\;i=3,4.$$

where σ(·) is the sigmoid function.

The final fused output $F_{\mathrm{fuse}}^{i}$ (i∈{3,4}) is computed using the following asymmetric weighted formula:

(21) $$F_{\mathrm{fuse}}^{i}=F_{\mathrm{RGB}}^{i}\cdot W^{i}+F_{T}^{i}\cdot \left(1-W^{i}\right),\;i=3,4.$$

**Figure 5:**
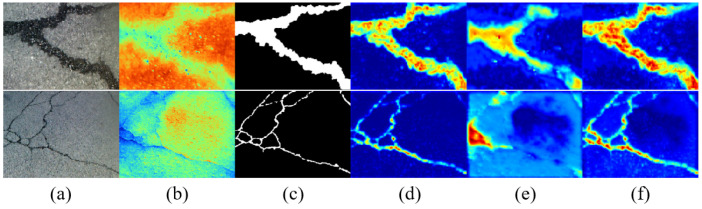
Comparison features of the AFF module before and after fusion: (**a**) original RGB images, (**b**) original thermal images, (**c**) label images, (**d**) RGB features before fusion, (**e**) thermal features before fusion, and (**f**) fused features.

This attention-driven mechanism reflects the theory of selective response in deep learning, effectively boosting the model’s ability to identify crack-related regions across modalities. Figure 5 presents the visualization of the AFF module before and after fusion on the asphalt pavement crack detection dataset.

### 2.2. Structure-Guided Crack Segmentation

To reconstruct high-resolution semantic features and recover spatial details lost during the encoding process, a hierarchical decoder with edge-guided fusion is proposed. The decoder adopts a symmetric top–down architecture composed of four upsampling stages. Each stage consists of an upsampling convolution module followed by a nonlinear transformation block and, where applicable, a skip connection that fuses encoder features of the corresponding scale. This progressive decoding process gradually restores low-level textures and high-level semantics, enabling the precise spatial localization of cracks.

At each stage, the decoder outputs an intermediate feature map with a different semantic abstraction level. These decoded features are then passed to a dedicated fusion and prediction head consisting of four parallel branches. Each branch first reduces the channel dimension of the decoded feature via a convolutional projection and then upsamples the feature map to the original resolution. A corresponding edge prediction branch then generates a crack edge probability map $P_{\mathrm{Edge}}^{i}$. The predicted edge map is compared with the ground truth edge map obtained via the Canny operator, and a mean squared error (MSE) loss is computed. The resulting edge losses from multiple layers are normalized to compute layer-wise fusion weights, which are concatenated with the upsampled feature and processed by a 1×1 convolution to produce the segmentation predictions $P_{\mathrm{seg}}^{i}$. This process is applied independently across all four levels, resulting in candidate segmentation outputs $P_{\mathrm{seg}}^{i}$ and $P_{\mathrm{Edge}}^{i}$ (i∈{1,2,3,4}).

To guide the segmentation process with fine-grained structural awareness, an edge-aware supervision strategy is employed. The ground-truth edge map is obtained using the Canny operator and transformed into a two-channel binary mask representing edge and non-edge regions. The predicted edge maps are supervised using the mean squared error (MSE) loss. Let $L^{i}$ denote the weighted loss at the i-th level, which is computed as follows:

(22) $$L^{i}=\frac{1}{L_{\mathrm{MSE}}^{i}},\;i=1,2,3,4.$$

(23) $$L_{\mathrm{MSE}}^{i}=\frac{1}{N}\sum_{k=1}^{N}{\left(P_{\mathrm{Edge}}^{\mathrm{ki}}-Y_{\mathrm{Edge}}^{k}\right)}^{2},\;i=1,2,3,4.$$

where $L_{\mathrm{MSE}}^{i}$ denotes the MSE loss of the crack edge prediction result at the i-th level, N is the number of training samples, $P_{\mathrm{Edge}}^{\mathrm{ki}}$ represents the predicted edge result of the i-th level for the k-th sample, and $Y_{\mathrm{Edge}}^{k}$ is the ground truth edge label of the k-th sample.

The weighted loss values of all levels are summed to obtain the total weighted loss.

(24) $$L_{t}=\sum_{i=1}^{n}L^{i},\;i=1,2,3,4.$$

Then, the weight of the crack prediction result at the i-th level is calculated:

(25) $$\omega_{i}=\frac{L^{i}}{L_{t}},\;i=1,2,3,4.$$

After that, for the input RGB image and thermal image of the object to be detected, the final crack prediction result of the object to be detected is obtained based on the weights:

(26) $$P=\sum_{i=1}^{n}\omega_{i}\cdot P_{\mathrm{seg}}^{i},\;n=4.$$

**Figure 6:**
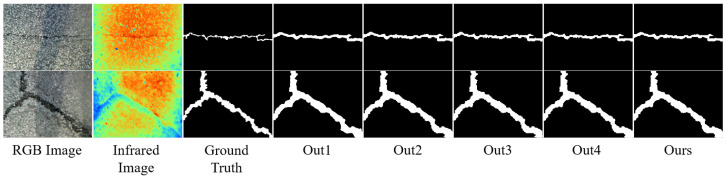
Visualization before and after adaptive fusion. Out1, Out2, Out3 and Out4 represent the corresponding outputs of the four decoder layers.

The visualization comparison before and after adaptive fusion is illustrated in Figure 6. This edge-guided weighting mechanism ensures that feature maps with more accurate edge predictions contribute more significantly to the final result. The combination of hierarchical decoding, skip connections, edge prediction, and adaptive fusion allows the network to effectively capture both semantic context and spatial precision, which is essential for accurate crack detection in complex environments.

### 2.3. Loss Function

To enhance the accuracy and robustness of crack segmentation under complex surface conditions, a hybrid loss function is designed to guide both semantic prediction and boundary localization. The proposed loss function integrates three complementary components: an edge-guided loss, a multi-scale focal loss, and an adaptive fusion loss. These components jointly supervise the hierarchical outputs of the decoder and enforce consistency between segmentation and edge representations.

#### 2.3.1. Edge-Guided Loss

Each intermediate decoded feature is associated with a corresponding edge prediction map, which is supervised using the mean squared error (MSE) loss, and its formulation is provided in Section 2.2. The ground-truth edge map is generated from the segmentation label using a Canny operator and then split into two binary channels representing edge and non-edge regions.

The total edge loss is the sum over all levels:

(27) $$L_{\mathrm{Edge}}=\sum_{i=1}^{n}L_{\mathrm{MSE}}^{i},\;n=4.$$

#### 2.3.2. Multi-Scale Focal Loss

To address the common issue of class imbalance in crack segmentation, especially when cracks occupy only a small portion of the image, the focal loss is employed at each level of the segmentation outputs. The formula for the focal loss is as follows:

(28) $$\begin{array}{c} L_{\mathrm{Focal}}^{\left(i\right)}=-\frac{1}{N}\sum_{k=1}^{N}[\alpha {\left(1-p_{k}^{\left(i\right)}\right)}^{\gamma }y_{k}^{\left(i\right)}\log\left(p_{k}^{\left(i\right)}\right)\\ \;\;\;\;\;\;\;\;\;\;\;\;\;\;\;\;\;\;\;\;\;\;\;\;\;\;\;\;\;\;\;\;\;\;\;\;\;\;\;\;+\left(1-\alpha \right){\left(p_{k}^{\left(i\right)}\right)}^{\gamma }\left(1-y_{k}^{\left(i\right)}\right)\log\left(1-p_{k}^{\left(i\right)}\right)],\;\mathrm{for}\;i=1,2,3,4. \end{array}$$

where N is the number of training samples, α is the weighting factor, γ is the focusing parameter, $P_{k}$ is the final predicted crack result of the k-th sample, and $Y_{k}$ is the corresponding ground truth label.

The segmentation loss for each level is computed as shown below:

(29) $$L_{\mathrm{seg}}^{i}=L_{\mathrm{Focal}}^{i}\left(P_{\mathrm{seg}}^{i},Y\right),\;i=1,2,3,4.$$

The aggregated segmentation loss across all hierarchical outputs is as follows:

(30) $$L_{\mathrm{seg}}=\sum_{i=1}^{n}L_{\mathrm{seg}}^{i},\;n=4.$$

#### 2.3.3. Adaptive Fusion Loss

The fusion output P obtained from Section 3.3 is further supervised by using focal loss:

(31) $$L_{\mathrm{fuse}}=L_{\mathrm{Focal}}\left(P,Y\right).$$

#### 2.3.4. Total Loss

The total loss function is defined as shown below:

(32) $$L_{\mathrm{total}}=L_{\mathrm{Edge}}+L_{\mathrm{seg}}+L_{\mathrm{fuse}}.$$

This composite loss ensures that edge structures are preserved, segmentation outputs are robust to class imbalance, and multi-level predictions are effectively fused under structural guidance.

## 3. Experiment

In this section, extensive experiments are performed on public datasets to verify the performance of the proposed method.

### 3.1. Dataset

Experimental evaluations were performed on two publicly available datasets: the asphalt pavement crack detection dataset and the Crack900 dataset. The former consists of crack imagery collected from asphalt pavement surfaces, whereas the latter is composed of crack samples acquired from masonry wall structures.

**Asphalt pavement crack detection dataset** (available at: https://github.com/lfangyu09/IR-Crack-detection (accessed on 1 December 2024)): This dataset is a public benchmark specifically curated for crack segmentation based on Infrared Thermography [42]. It is a RGB-T asphalt pavement crack segmentation dataset and includes four categories of images: RGB, thermal, RGB-T fused images (created by combining both modalities equally using IR-Fusion™ technology), and manually annotated ground truth masks created using Photoshop. Each category contains 448 images, all with a uniform resolution of 640 × 480 pixels. For experimental purposes, the dataset is divided into two subsets: 358 samples for training and 90 for testing. The dataset was collected across three different time periods, including morning (8:00 a.m.), noon (12:00 p.m.), and dusk (5:00 p.m.). The maximum pavement temperature was nearly identical to the daily maximum temperature, while the temperature at dusk was slightly higher than in the morning. At noon, some crack regions in the images exhibited similar temperatures to the background, whereas the distinction between cracks and background was more pronounced in dusk and morning images. Since shadows from guardrails and trees may compromise image quality, all images were captured on sidewalks without guardrails or trees to minimize environmental interference.

**Crack900 dataset** (available at: https://data.mendeley.com/datasets/kz84t85z66/1 (accessed on 1 August 2025)): This dataset comprises RGB, thermal, and RGB-T fused images with each category containing 1081 images [43]. The dataset was randomly partitioned into a training set (80%) and a test set (20%), resulting in 731 image groups for training and 183 image groups for validation. All images have a resolution of 288 × 384 pixels. Additionally, data augmentation techniques—including RandomFlip, RandomRotate, and RandomCrop—were applied to expand the dataset. The dataset was collected from masonry scenes containing cracks. Masonry structures are characterized by more complex textures and noise caused by mortar joints, which often resemble cracks and can easily lead to false detections. Training and testing solely on images with highly regular brick shapes, sizes, and colors may result in high accuracy within such constrained scenarios but cause a significant performance drop when applied to other environments. To avoid this limitation, the dataset was collected in scenes with diverse brick patterns, ensuring greater variability and robustness in crack segmentation.

### 3.2. Implementation Details

The proposed method is implemented with the PyTorch framework (version 1.12.1) where the Adam optimizer and a weight decay parameter of $1\times 10^{-4}$ are used. The training is achieved with the batch size of 4 over the course of 200 epochs. All experiments are implemented on a server equipped with an Nvidia GeForce GTX 3090 GPU. The settings of experimental parameters are presented in Table 1. The total training time of our model is approximately 200 min; however, based on multiple experimental trials, the model achieves optimal performance within 170 min. The inference process for obtaining both visual results and accuracy metrics takes approximately 30 s. The overall efficiency demonstrates strong applicability for practical crack detection tasks.

**Figure 7:**
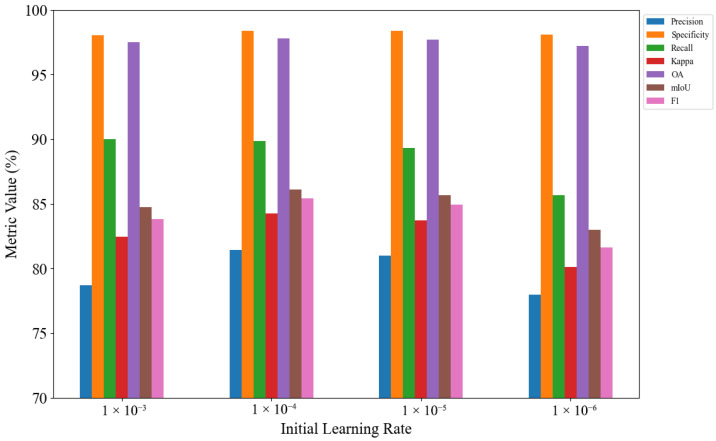
Initial learning rate analysis.

To investigate the impact of the initial learning rate on model performance, we conducted a set of controlled experiments on the asphalt pavement crack detection dataset by varying the learning rate while keeping other hyperparameters fixed. Specifically, four different values were tested: $1\times 10^{-3}$, $1\times 10^{-4}$, $1\times 10^{-5}$, $1\times 10^{-6}$. The corresponding segmentation performance is illustrated in Figure 7.

When the initial learning rate was set to $1\times 10^{-4}$, the model achieved the best overall performance. In contrast, lower learning rates such as $1\times 10^{-5}$ and $1\times 10^{-6}$ led to sub-optimal convergence, which is possibly due to insufficient gradient updates that slow down learning and risk becoming stuck in local minima. On the other hand, a higher learning rate of $1\times 10^{-3}$ resulted in unstable optimization with a relatively lower accuracy and F1-score, which may be attributed to overshooting the optimal solution during backpropagation.

The learning rate controls the step size of gradient descent—too small a value reduces convergence efficiency, while too large a value tends to cause unstable training. Therefore, choosing $1\times 10^{-4}$ as the initial learning rate achieves a good balance between stability and convergence speed, ultimately resulting in superior generalization performance.

**Figure 8:**
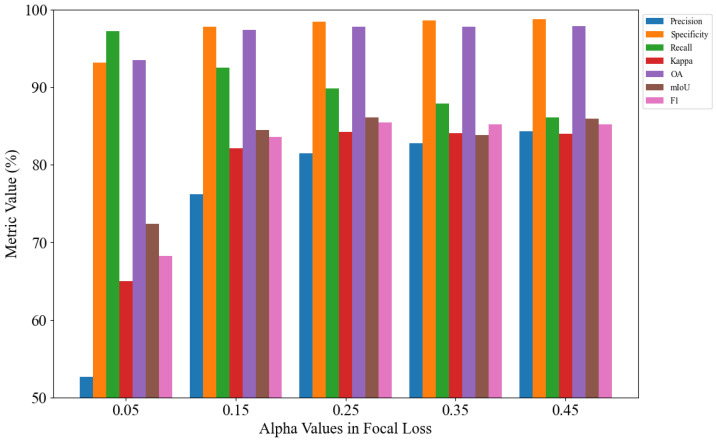
Focal loss alpha analysis.

**Figure 9:**
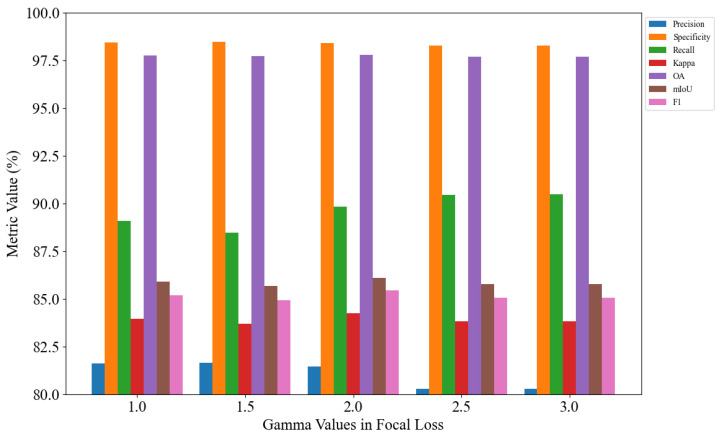
Focal loss gamma analysis.

An experimental analysis is conducted on the parameter settings of α and γ in the focal loss. First, γ is fixed at 2.0, and α is set to 0.05, 0.15, 0.25, 0.35, and 0.45 for five separate experiments, as illustrated in Figure 8. Subsequently, α is fixed at 0.25, and γ is set to 1.0, 1.5, 2.0, 2.5, and 3.0 for another five experiments, as shown in Figure 9.

**Figure 10:**
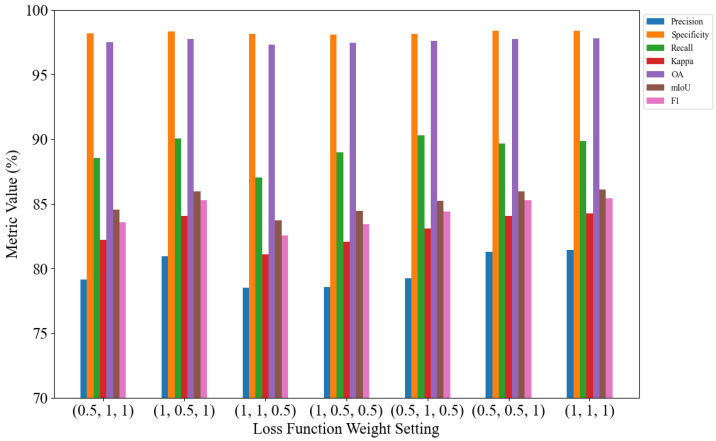
Loss function weight analysis.

The weights of the three loss components were systematically evaluated using seven different combinations: (0.5, 1, 1), (1, 0.5, 1), (1, 1, 0.5), (1, 0.5, 0.5), (0.5, 1, 0.5), (0.5, 0.5, 1), and (1, 1, 1). The corresponding results are presented as a bar chart in Figure 3. As shown in Figure 10, the model performs optimally when the weights of the three components of the loss function are set to (1, 1, 1).

As shown in Figure 8 and Figure 9, the model achieves optimal performance when α is set to 0.25 and γ is set to 2.0. Therefore, setting α to 0.25 and γ to 2.0 yields the best segmentation performance.

Ablation experiments are conducted on the three loss components to validate their individual contributions. The results are presented in Table 2.

As shown in Table 2, removing any component of the loss function results in a decline in the segmentation performance of the model, indicating that each loss component is essential.

### 3.3. Evaluation Metrics

Crack detection and segmentation belong to a binary classification problem: common evaluation metrics include precision, specificity, recall, kappa, overall accuracy (OA), mean intersection over union (mIoU) and F1-score. Precision refers to the ratio of true positive samples to all predicted positive samples, reflecting the false positive rate. Specificity refers to the proportion of true negative samples correctly predicted out of all actual negative samples. Recall refers to the ratio of true positive samples to all true positive samples, reflecting the false negative rate. Kappa is an index used to measure the consistency between model prediction results and actual classification results, reflecting the classification performance. IoU is the intersection over union of the predicted map and the ground truth map, while mIoU is the mean IoU across all categories. F1-score is the harmonic mean of precision and recall, which takes into account both false positive and false negative rates, and it can better reflect the detection capability of the method used. Overall accuracy reflects the proportion of correctly classified pixels to the total pixels. Higher values for these metrics indicate superior segmentation performance. Since OA, F1 and mIoU can better reflect segmentation recognition capability, this paper mainly refers to these three metrics to compare the effectiveness of various methods. The expressions for each metric are as follows:

(33) $$\mathrm{Precision}=\frac{\mathrm{TP}}{\mathrm{TP}+\mathrm{FP}},$$

(34) $$\mathrm{Specificity}=\frac{\mathrm{TN}}{\mathrm{TN}+\mathrm{FP}},$$

(35) $$\mathrm{Recall}=\frac{\mathrm{TP}}{\mathrm{TP}+\mathrm{FN}},$$

where TP represents the number of true positives (correctly predicted positive samples), FN represents the number of false negatives (incorrectly predicted positive samples), FP represents the number of false positives (incorrectly predicted negative samples), and TN represents the number of true negatives (correctly predicted negative samples).

(36) $$\mathrm{Kappa}=\frac{p_{0}-p_{e}}{1-p_{0}},$$

where $p_{0}$ is the accuracy rate, and $p_{e}$ is the sum of the “product of actual and predicted quantities” for all categories divided by the “square of the total number of samples”.

(37) $$\mathrm{OA}=\frac{\mathrm{TP}+\mathrm{TN}}{\mathrm{TP}+\mathrm{FN}+\mathrm{FP}+\mathrm{TN}},$$

(38) $$\mathrm{mIoU}=\frac{1}{n}\sum_{l=1}^{n}\frac{{\mathrm{TP}}_{l}}{{\mathrm{TP}}_{l}+{\mathrm{FP}}_{l}+{\mathrm{FN}}_{l}},$$

where n represents the number of categories.

(39) $$F1=\frac{2\times \mathrm{Precision}\times \mathrm{Recall}}{\mathrm{Precision}+\mathrm{Recall}}.$$

### 3.4. Ablation Study

**Figure 11:**
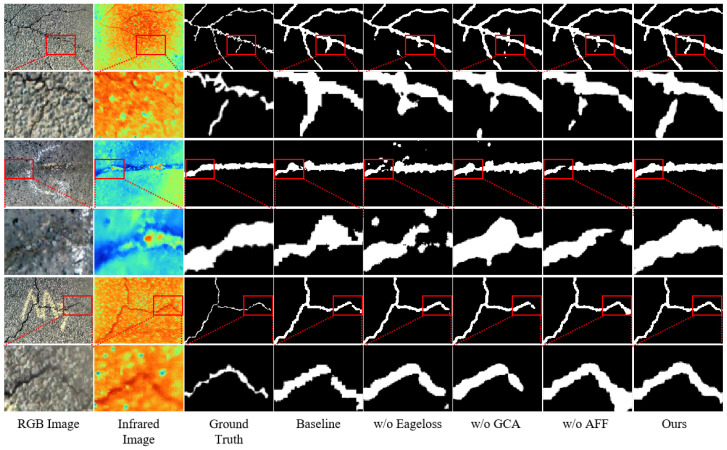
Visualization of ablation experiments. The images in rows 2, 4, 6, and 8 present locally magnified views of the respective red-boxed regions in the corresponding images from rows 1, 3, 5, and 7.

We conducted ablation studies to validate the effectiveness of each component on the asphalt pavement crack detection dataset with each fusion module in the model assessed individually. The edge loss function was also evaluated separately, and the results can be found in Table 3, while the visualization results are presented in Figure 11. To observe the differences more clearly, the image was subjected to partial enlargement.

### 3.5. Comparison Experiments with Different Methods

To validate the effectiveness of the proposed method, we compared SPMFNet with eight advanced approaches on the two datasets. Among these, U-net [6] and Crackformer-II [11] use only RGB images or infrared images. CAINet [44], MCNet [45], SFAF-MA [46], SGFNet [47], GMNet [30] and IRFusionFormer [48] employ both infrared and RGB images as inputs.

**Figure 12:**
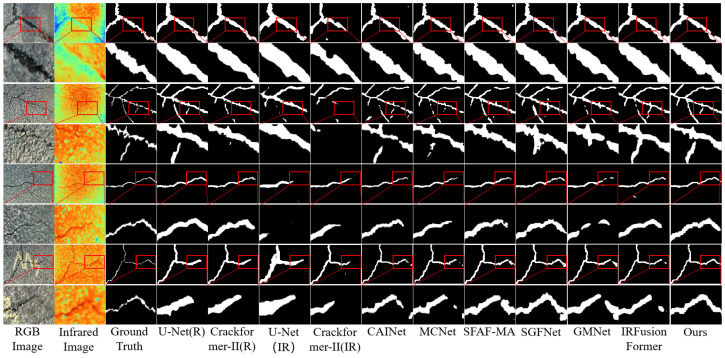
Comparison of crack segmentation results from nine models on the asphalt pavement crack detection dataset. The images in rows 2, 4, 6, and 8 present locally magnified views of the respective red-boxed regions in the corresponding images from rows 1, 3, 5, and 7.

*(1) Comparison on the asphalt pavement crack detection dataset:* The results in Table 4 indicate that the RGB-T integrated model outperforms the model that uses only RGB images, both of which are superior to the model that uses only infrared images on the asphalt pavement crack detection dataset. As shown in Figure 12, on the test dataset, the proposed SPMFNet achieved the best results in terms of six evaluation metrics, outperforming the second best model SGFNet in precision, specificity, recall, kappa, OA, mIoU, and F1, at 3.83%, 0.39%, 1.36%, 1.28%, 0.52%, 1.45%, and 1.92%, respectively. To observe the differences more clearly, the image has been subjected to partial enlargement. From the figures, it can be observed that SPMFNet can still produce accurate segmentation results under severe noise conditions, such as shadows, paint-like interference, and large-area speckles. For fine cracks, SPMFNet can also achieve high-precision results.

**Figure 13:**
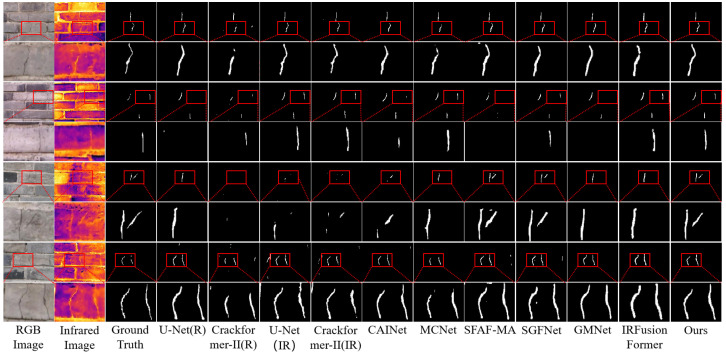
Comparison of crack segmentation results from nine models on the Crack900 dataset. The images in rows 2, 4, 6, and 8 present locally magnified views of the respective red-boxed regions in the corresponding images from rows 1, 3, 5, and 7.

*(2) Comparison on the Crack900 dataset:* As presented in Table 5, the proposed SPMFNet achieves the most competitive overall performance on the masonry crack dataset Crack900. As illustrated in Figure 13, SPMFNet demonstrates significantly reduced false positives and missed detections compared to other state-of-the-art network models, highlighting its superior detection capability. From the figures, it can be seen that for fine cracks in masonry scenarios, SPMFNet can accurately segment and restore the true morphology of the cracks.

## 4. Conclusions

This paper presents an end-to-end edge-guided progressive multi-modal segmentation network for crack detection, which effectively integrates RGB and thermal infrared information through a carefully designed fusion strategy. In the first two encoder layers, we use a GCA module to adaptively fuse shallow features, preserving fine spatial details while reducing noise. In the deeper layers, the AFF module applies local and global attention to weight multi-level features, ensuring a robust fusion of RGB and thermal infrared information. The GCA module enhances shallow feature fusion, while the AFF module refines deeper fusion, enabling the model to capture both spatial details and semantic context for high-precision crack segmentation. The Squeeze-and-Excitation Attention and AFF modules used in the paper are existing components. However, by combining them with other components in a novel technical manner, we ultimately achieved relatively good feature fusion effects in the progressive multi-modal feature fusion stage. By incorporating structural priors and edge-aware supervision, the proposed method achieves a high-precision segmentation of fine-grained crack structures and demonstrates strong robustness across diverse scenarios. The proposed method can compensate for the limitations of traditional RGB crack detection methods under adverse weather conditions. Moreover, it demonstrates superior capability in detecting crack edge shapes, which provides significant assistance for subsequent repair work. The experimental results validate its consistent superiority over existing approaches in both accuracy and reliability. For instance, the Transformer network can be incorporated into the model to capture long-range dependencies and contextual relationships between cracks, which is particularly useful for crack detection in large-scale or complex structural surfaces. Self-supervised learning, on the other hand, can improve the model’s adaptability under weak labeling conditions by learning useful representations from unlabeled data, thus reducing the reliance on manually annotated datasets. These advancements are expected to enhance the robustness of the model and improve its generalization ability, especially when dealing with small sample sizes and varying environmental conditions.

## Figures and Tables

**Table 1:** Experimental parameter settings.

Settings	SPMFNet
Optimizer	Adam
Learning Rate	$1\times 10^{-4}$
Epochs	200
Batch Size	4
Image Size	480 × 640/288 × 384

**Table 2:** Contribution analysis of loss function components. The bold entries in the table indicate the best results under each evaluation metric.

Variants	P	Spec	Rec	Kappa	OA	mIoU	F1
w/o loss1	76.15	97.73	**92.81**	82.25	97.37	84.55	83.66
w/o loss2	55.03	95.32	73.30	59.51	93.72	69.61	62.87
w/o loss3	74.71	97.83	82.15	76.47	96.69	80.38	78.25
SPMFNet	**81.45**	**98.40**	89.84	**84.23**	**97.78**	**86.10**	**85.44**

**Table 3:** Quantitative results (%) of the evaluation of GCA, AFF, and edge-guided loss in SPMFNet. The bold entries in the table indicate the best results under each evaluation metric.

Variants	P	Spec	Rec	Kappa	OA	mIoU	F1
baseline	81.01	98.05	84.42	81.29	97.44	83.87	82.68
w/o EageLoss	80.71	98.39	86.42	82.13	97.52	84.49	83.47
w/o GCA	78.56	98.09	89.56	82.34	97.47	84.63	83.70
w/o AFF	80.07	98.29	88.07	82.56	97.55	84.81	83.88
SPMFNet	**81.45**	**98.40**	**89.84**	**84.24**	**97.78**	**86.10**	**85.44**

**Table 4:** Segmentation performance (%) for different methods on the asphalt pavement crack detection dataset. The bold entries in the table indicate the best results under each evaluation metric.

Type	Model	P	Spec	Rec	Kappa	OA	mIoU	F1
RGB	U-Net [6]	78.24	98.06	89.07	81.91	97.42	84.31	83.30
RGB	Crackformer-II [11]	80.07	98.28	88.38	82.70	97.56	84.92	84.42
T	U-Net [6]	54.61	94.90	78.53	61.09	91.73	70.42	64.44
T	Crackformer-II [11]	67.82	97.83	84.50	70.98	94.98	70.24	62.76
RGB-T	CAINet [44]	81.06	**98.51**	81.85	81.00	97.30	82.82	81.46
RGB-T	MCNet [45]	78.95	98.24	84.63	81.06	97.25	83.13	82.08
RGB-T	SFAF-MA [46]	78.85	97.62	85.97	83.61	97.31	85.19	82.54
RGB-T	SGFNet [47]	77.62	98.01	88.48	82.96	97.26	84.65	83.52
RGB-T	GMNet [30]	78.28	98.09	82.39	81.44	97.17	82.44	82.98
RGB-T	IRfusionFormer [48]	79.57	98.27	86.23	81.36	97.40	83.91	82.77
RGB-T	SPMFNet	**81.45**	98.40	**89.84**	**84.24**	**97.78**	**86.10**	**85.44**

**Table 5:** Segmentation performance (%) for different methods on the Crack900 dataset. The bold entries in the table indicate the best results under each evaluation metric.

Type	Model	P	Spec	Rec	Kappa	OA	mIoU	F1
RGB	U-Net [6]	56.60	99.77	57.62	56.88	99.55	69.76	57.10
RGB	Crackformer-II [11]	42.77	99.68	45.92	43.99	99.41	63.93	44.29
T	U-Net [6]	63.54	99.81	**64.96**	64.06	99.63	73.47	64.24
T	Crackformer-II [11]	59.16	99.77	64.37	61.45	99.59	72.07	61.65
RGB-T	CAINet [44]	58.98	99.81	52.31	55.23	99.57	68.96	55.45
RGB-T	MCNet [45]	64.13	99.83	59.41	61.49	99.62	72.11	61.68
RGB-T	SFAF-MA [46]	53.31	99.72	62.01	57.09	99.52	69.85	57.33
RGB-T	SGFNet [47]	47.06	99.64	62.64	53.47	99.45	68.09	53.74
RGB-T	GMNet [30]	59.02	99.78	59.92	59.26	99.58	70.95	59.47
RGB-T	IRfusionFormer [48]	42.11	99.56	61.94	49.83	99.37	66.41	50.14
RGB-T	SPMFNet	**66.63**	**99.83**	64.89	**65.58**	**99.65**	**74.31**	**65.75**

## Data Availability

The datasets used in this study can be downloaded from the URLs in their official websites. The code is accessible from the corresponding author upon reasonable request.

## References

[1] Wang H., Wu G., Liu Y. (2025). Efficient generative-adversarial U-Net for multi-organ medical image segmentation. J. Imaging.

[2] Dais D., Bal I.E., Smyrou E., Sarhosis V. (2021). Automatic crack classification and segmentation on masonry surfaces using convolutional neural networks and transfer learning. Autom. Constr..

[3] Tran T.S., Nguyen S.D., Lee H.J., Tran V.P. (2023). Advanced crack detection and segmentation on bridge decks using deep learning. Constr. Build. Mater..

[4] Wang H., Li Y., Dang L.M., Lee S., Moon H. (2021). Pixellevel tunnel crack segmentation using a weakly supervised annotation approach. Comput. Ind..

[5] Han C., Yang H., Ma T., Wang S., Zhao C., Yang Y. (2024). Crackdiffusion: A two-stage semantic segmentation framework for pavement crack combining unsupervised and supervised processes. Autom. Constr..

[6] Long J., Shelhamer E., Darrell T. Fully convolutional networks for semantic segmentation. Proceedings of the IEEE Conference on Computer Vision and Pattern Recognition (CVPR).

[7] Ronneberger O., Fischer P., Brox T. (2015). U-Net: Convolutional networks for biomedical image segmentation. Proceedings of the Medical Image Computing and Computer-Assisted Intervention—MICCAI 2015.

[8] Chen L.C., Zhu Y., Papandreou G., Schroff F., Adam H. Encoder-decoder with atrous separable convolution for semantic image segmentation. Proceedings of the European Conference on Computer Vision (ECCV).

[9] Du Y., Zhang X., Li F., Sun L. (2017). Detection of crack growth in asphalt pavement through use of infrared imaging. Transp. Res. Rec..

[10] Ma M., Lei Y., Liu Y., Yu H. (2024). An attention-based progressive fusion network for pixelwise pavement crack detection. Measurement.

[11] Liu H., Yang J., Miao X., Mertz C. (2023). CrackFormer network for pavement crack segmentation. IEEE Trans. Intell. Transp. Syst..

[12] Ren Y., Huang J., Hong Z., Lu W., Yin J., Zou L., Shen X. (2020). Image-based concrete crack detection in tunnels using deep fully convolutional networks. Constr. Build. Mater..

[13] Liu Y. (2025). DeepLabV3+ Based Mask R-CNN for Crack Detection and Segmentation in Concrete Structures. Int. J. Adv. Comput. Sci. Appl..

[14] Hou W., He J., Cui C., Zhong F., Jiang X., Lu L., Zhang J., Tu C. (2025). Segmentation refinement of thin cracks with minimum strip cuts. Adv. Eng. Inf..

[15] Li S., Gou S., Yao Y., Chen Y., Wang X. (2024). Physically informed prior and cross-correlation constraint for fine-grained road crack segmentation. Proceedings of the Chinese Conference on Pattern Recognition and Computer Vision (PRCV).

[16] Yoon H., Kim H.K., Kim S. (2025). PPDD: Egocentric crack segmentation in the port pavement with deep learning-based methods. Appl. Sci..

[17] Sun W., Liu X., Lei Z. (2025). Research on tunnel crack identification localization and segmentation method based on improved YOLOX and UNETR++. Sensors.

[18] Zhang Z., Zhuang Y., Song W., Wu J., Ye X., Zhang H., Xu Y., Shi G. (2025). ISTD-CrackNet: Hybrid CNN-transformer models focusing on fine-grained segmentation of multi-scale pavement cracks. Measurement.

[19] Wang L., Wu G., Tossou A.I.H.C.F., Liang Z., Xu J. (2025). Segmentation of crack disaster images based on feature extraction enhancement and multi-scale fusion. Earth Sci. Inform..

[20] Si J., Lu J., Zhang Y. (2025). An FCN-based segmentation network for fine linear crack detection and measurement in metals. Int. J. Struct. Integr..

[21] Wang Z., Zeng Z., Huang F., Sherratt R.S., Alfarraj O., Tolba A., Zhang J. (2025). A U-Net-like full convolutional pavement crack segmentation network based on multi-layer feature fusion. Int. J. Pavement Eng..

[22] Wang C., Liu H., An X., Gong Z., Deng F. (2025). DCNCrack: Pavement crack segmentation based on large-scaled deformable convolutional network. J. Comput. Civ. Eng..

[23] Guo X., Tang W., Wang H., Wang J., Wang S., Qu X. (2025). MorFormer: Morphology-aware transformer for generalized pavement crack segmentation. IEEE Trans. Intell. Transp. Syst..

[24] Zeng L., Zhang C., Cai S., Yan X., Wang S. (2025). Deep crack segmentation: A semi-supervised approach with coordinate attention and adaptive loss. Meas. Sci. Technol..

[25] Liang F., Li Q., Yu H., Wang W. (2025). CrackCLIP: Adapting vision-language models for weakly supervised crack segmentation. Entropy.

[26] Kütük Z., Algan G. Semantic segmentation for thermal images: A comparative survey. Proceedings of the IEEE/CVF Conference on Computer Vision and Pattern Recognition.

[27] Wang Z., Zhang H., Qian Z., Chen L. (2023). A complex scene pavement crack semantic segmentation method based on dual-stream framework. Int. J. Pavement Eng..

[28] Ha Q., Watanabe K., Karasawa T., Ushiku Y., Harada T. MFNet: Towards real-time semantic segmentation for autonomous vehicles with multi-spectral scenes. Proceedings of the 2017 IEEE/RSJ International Conference on Intelligent Robots and Systems (IROS).

[29] Zhang Q., Zhao S., Luo Y., Zhang D., Huang N., Han J. ABMDRNet: Adaptive-weighted bi-dTectional modality difference reduction network for RGB-T semantic segmentation. Proceedings of the 2021 IEEE/CVF Conference on Computer Vision and Pattern Recognition (CVPR).

[30] Zhou W., Liu J., Lei J., Yu L., Hwang J. (2021). GMNet: Graded-feature multilabel-learning network for RGB-thermal urban scene semantic segmentation. IEEE Trans. Image Process..

[31] Zhou W., Dong S., Xu C., Yaguan Q. Edge-aware guidance fusion network for RGB-thermal scene parsing. Proceedings of the AAAI Conference on Artificial Intelligence.

[32] Zhou W., Zhang H., Yan W., Lin W. (2023). MMSMCNet: Modal memory sharing and morphological complementary networks for RGB-T urban scene semantic segmentation. IEEE Trans. Circuits Syst. Video Technol..

[33] Yang Y., Shan C., Zhao F., Liang W., Han J. (2024). On exploring shape and semantic enhancements for RGB-X semantic segmentation. IEEE Trans. Intell. Veh..

[34] Wang Q., Yin C., Song H., Shen T., Gu Y. (2023). UTFNet: Uncertainty-guided trustworthy fusion network for RGB-Thermal semantic segmentation. IEEE Geosci. Remote Sens. Lett..

[35] Chen H., Wang Z., Qin H., Mu X. (2024). DHFNet: Decoupled hierarchical fusion network for RGB-T dense prediction tasks. Neurocomputing.

[36] Zhao G., Huang J., Peng T. Open-vocabulary RGB-Thermal semantic segmentation. Proceedings of the European Conference on Computer Vision.

[37] Liu C., Liu H., Ma H. (2026). Implicit alignment and query refinement for RGB-T semantic segmentation. Pattern Recognit..

[38] Liu J., Liu H., Xu X. (2025). MiLNet: Multiplex interactive learning network for RGB-T semantic segmentation. IEEE Trans. Image Process..

[39] Tang L., Yuan J., Ma J. (2022). Image fusion in the loop of high-level vision tasks: A semantic-aware real-time infrared and RGB image fusion network. Inform. Fusion.

[40] Hu J., Shen L., Sun G. Squeeze-and-excitation networks. Proceedings of the IEEE Conference on Computer Vision and Pattern Recognition.

[41] Dai Y., Gieseke F., Oehmcke S., Wu Y. Attentional feature fusion. Proceedings of the IEEE/CVF Winter Conference on Applications of Computer Vision.

[42] Liu F., Liu J., Wang L. (2022). Asphalt pavement crack detection based on convolutional neural network and infrared thermography. IEEE Trans. Intell. Transport. Syst..

[43] Huang H., Cai Y., Zhang C. (2024). Crack detection of masonry structure based on thermal and visible image fusion and semantic segmentation. Autom. Constr..

[44] Lv Y., Liu Z., Li G. (2024). Context-aware interaction network for RGB-T semantic segmentation. IEEE Trans. Multimedia.

[45] Guo X., Liu T., Mou Y., Chai S., Ren B., Wang Y. (2025). Transferring prior thermal knowledge for snowy urban scene semantic segmentation. IEEE Trans. Intell. Transp. Syst..

[46] He X., Wang M., Liu T., Zhao L., Yue Y. (2023). SFAF-MA: Spatial feature aggregation and fusion with modality adaptation for RGB-thermal semantic segmentation. IEEE Trans. Instrum. Meas..

[47] Wang Y., Li G., Liu Z. (2023). SGFNet: Semantic-guided fusion network for RGB-thermal semantic segmentation. IEEE Trans. Circuits Syst. Video Technol..

[48] Xiao R., Chen X. (2024). IRFusionFormer: Enhancing Pavement Crack Segmentation with RGB-T Fusion and Topological-Based Loss. arXiv.

